# Photocatalytic Gas-Phase Hydrogen Sulfide Removal Using Mo Cocatalyst: Implementation of Counter-Poisoning Photocycle

**DOI:** 10.1021/acsomega.4c09395

**Published:** 2025-01-29

**Authors:** Tomofumi Katayama, Morio Nagata

**Affiliations:** Department of Industrial Chemistry, Graduate School of Engineering, Tokyo University of Science, 6-3-1 Niijuku, Katsushika-ku, Tokyo 125-8585, Japan

## Abstract

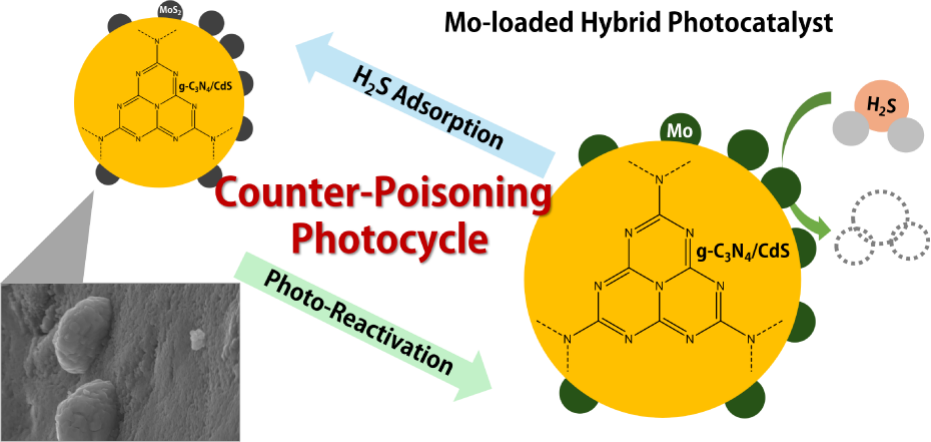

Hydrogen sulfide is a toxic gas known for its foul odor and health risks, even at significantly low concentrations. Although its decomposition at high concentrations is common in industrial processes, decomposing it at low concentrations is difficult. One of the difficulties is that sulfate ions generated during the reaction would poison the catalyst and reduce efficiency. Here, we show the gas-phase decomposition of low-concentration hydrogen sulfide using a high-activity photocatalyst, and to counter the poisoning problem, molybdenum is introduced through a novel photosupporting method that utilizes the characteristics of photocatalysts. In this study, we demonstrate a novel molybdenum-loaded catalyst for gas-phase photocatalytic hydrogen sulfide decomposition and its high performance: zero-out of 10 ppm of hydrogen sulfide. Moreover, the catalyst can be regenerated through photocatalytic reduction, keeping decomposing hydrogen sulfide to nearly odorless levels. The study provides a simple and sustainable photocatalytic process for removing low-concentration hydrogen sulfide, effectively preventing catalyst poisoning and enabling catalyst regeneration; thus, this suggests enhancing air quality and reducing health risks associated with this toxic gas in industrial and urban environments.

## Introduction

Considering the advancement of industrial technology and the development of cities, odor problems are a global concern. These odors are caused by organic volatiles from domestic wastewater, garbage, and wall materials in cities and pollution from exhaust and wastewater generated in factories and power plants. These gases are usually heavier than air, persisting in the environment and potentially causing long-term damage over a wide area from the source, as they can be perceived by the sense of smell, even at significantly low concentrations.

Hydrogen sulfide is a noxious, heavier-than-air gas with a rotten-egg odor. High concentrations of the gas are lethal, with levels as low as 2 ppm causing adverse effects such as dizziness, headaches, and tearing.^1^ Naturally occurring hydrogen sulfide in volcanic regions has concentrations of 0.1–100 ppm and has caused asphyxiation accidents in many parts of the world.^2^ Hydrogen sulfide in urban areas is mainly generated from sewage ditches. Industrially, hydrogen sulfide is produced in geothermal power plants, oil refineries, and sulfuric acid production plants and is released into the atmosphere after being removed to a certain extent by treatment methods. In the U.S., according to the Occupational Safety and Health Administration standards, factories and other facilities are designed to meet a standard of less than 20 ppm^3^ at the exhaust outlet; conversely, hydrogen sulfide emissions in Japan are regulated by law and must be disposed of at a concentration of less than 10 ppm at the outlet and 0.2 ppm at the site boundary.^4^ However, the odor-perception threshold concentration of hydrogen sulfide is 0.008 ppm,^5^ which complicates achieving complete odor control despite legal compliance.

Various industrial methods for managing hydrogen sulfide, including the Claus method, desulfurization by base absorption, and total underground reduction, have been utilized. The Claus method involves the reaction of hydrogen sulfide gas with oxygen under high-temperature and high-pressure conditions to obtain molten sulfur. This method increases the conversion rate by repeating the catalytic reaction and reducing the feed; however, it requires a high concentration of hydrogen sulfide (approximately 25%) in the feed, which is poisoned by the sulfur component and deteriorates. Hence, it is unsuitable for complete removal, generating approximately 100 ppm of hydrogen sulfide exhaust,^6^ with about an 80% conversion rate of sulfur oxide.

Base desulfurization involves the absorption of hydrogen sulfide by iron chloride and potassium carbonate, eventually leading to the fixation of the sulfur component.^7−9^ This method is classically used for the recovery of the sulfur source in sulfuric acid production and as a byproduct treatment in the production of wrecked coal gas for coke production; however, its recovery efficiency is not high, and approximately 50% of the sulfur content is released into the atmosphere as hydrogen sulfide.

In the total reduction method, mainly used in geothermal power plants, such as the Puna Power Plant in Hawaii, steam containing high concentrations of hydrogen sulfide is pressurized, dissolved in pumped hot water, and returned to the ground using a reduction well.^10^ Although this system is a comprehensive solution to groundwater depletion, a vital issue in geothermal power plants apart from odor control, it is not a versatile system owing to several challenges such as compressor corrosion, scaling, backflow of reduced gas, and failure of hot water to enter the reduction wells.

As indicated above, existing hydrogen sulfide treatment technologies are primarily used to treat high concentrations of hydrogen sulfide. They are limited by excessive costs, rapid process degradation, and hydrogen sulfide exhaust concentrations of approximately 10 ppm, rendering them unsuitable for implementation as odor control measures.^11^

Environmental remediation using photocatalysis is a widely studied technology that is gaining attention owing to its efficiency, low cost, and safety. Photocatalytic reactions have been used to decompose various substances and obtain decomposition products since Fujishima and Honda discovered a water-splitting reaction using titanium dioxide.^12^ Studies regarding hydrogen production,^13,14^ carbon dioxide reduction,^15^ and water purification^16^ are well-known. Canela et al. reported^17^ the decomposition of hydrogen sulfide using titanium dioxide, which was also reported by several other studies since then. Building on these studies, we reported the photocatalytic degradation of hydrogen sulfide at low concentrations under UV light irradiation using anatase/TiO_2_ (B) nanotubes^18^ and under visible light irradiation using a carbon nitride/CdS composite photocatalyst,^19^ which had not been achieved thus far. However, due to the accumulation and coating of sulfide and sulfate compounds in hydrogen sulfide degradation, the effect of poisoning was reportedly significant based on previous studies. This study proposes a new photocatalytic synthesis method to overcome poisoning and utilize photocatalytic devices that can operate more efficiently and longer. The research proposes a new photocatalytic synthesis method to overcome poisoning and utilize photocatalytic devices with higher efficiency and more prolonged operation.

Graphite-like carbon nitride and cadmium sulfide used in previous studies were utilized as photocatalytic substrates for the gas-phase decomposition of hydrogen sulfide. Graphite-like carbon nitride is a photocatalytic material with a two-dimensional planar structure and a band gap corresponding to the visible light range.^20^ This material is obtained via calcination of relatively inexpensive organic compounds such as urea, melamine, thiourea, and Dicyandiamide, and its low cost and simple mass synthesis are advantageous compared to other photocatalysts. Conversely, cadmium sulfide is synthesized via precipitation^21,22^ or solvothermal synthesis.^23^ It has a high photocatalytic activity; however, it is limited by autoxidative degradation. Combining these two photocatalysts to suppress electron recombination and improve reaction efficiency, we successfully achieved hydrogen sulfide decomposition in visible light up to the detection limit for the first time.

In this study, in addition to the photocatalytic substrate, molybdenum was introduced as a metal cocatalyst to suppress poisoning by the sulfur components, particularly sulfate ions, which are products of hydrogen sulfide decomposition. To synthesize this catalyst, we propose an easy and stable method for supporting molybdenum metal nanoparticles as an auxiliary catalyst, utilizing the photocatalytic activity of the base material. The thiourea-driven carbon nitride/cadmium sulfide/molybdenum photocatalyst (TCM) retains the high activity of the previously reported visible light-driven photocatalyst for decomposing low-concentration hydrogen sulfide vapor phase. It achieved efficient decomposition under visible light at extremely low concentrations, with superior toxicity tolerance and long-term degradation performance compared to earlier examples. The results also show that the photocatalyst can be regenerated to maintain and improve its performance. The photocatalyst developed in this study can decompose low concentrations of hydrogen sulfide to nearly odorless levels without additional energy administered under the same level of light irradiation as natural light. It can be used for a long duration with high cyclic stability if the catalyst is regenerated.

TCM is not only capable of maintaining a long-term high performance and catalytic activity by preventing catalyst poisoning and performance degradation owing to sulfides, as previously reported, but it can also maintain a high hydrogen sulfide decomposition performance by partially transforming the molybdenum particles into molybdenum sulfide,^24^ which demonstrates photocatalytic activity. This study focuses on the chemical transformation of molybdenum and the high performance of hydrogen sulfide decomposition.

The proposed photocatalytic method has high activity at low concentrations and cycle stability compared to existing industrial methods for hydrogen sulfide decomposition. It can be applied to existing processes using the adding-on method to solve the hydrogen sulfide odor problem while improving the overall performance of the process.

## Experimental Section

### Materials

Sodium sulfide nonahydrate (99%) and ammonium molybdate tetrahydrate (>99%) were purchased from Wako Pure Chemical Industries, Ltd., Japan. Thiourea (>98%), ethanol (99.5%), sodium hydroxide (>97%), cadmium nitrate tetrahydroxide (>97%), and methanol (99.5%) was purchased from Kanto Chemical Co. Ltd., Japan. All chemicals were used without further purification.

### Synthesis of CN/CdS Photocatalyst

The tCN/CdS photocatalyst was prepared using the same process we have previously studied. Namely, 20 g of thiourea was placed in an alumina crucible and calcined in an electric furnace under air at a firing temperature of 550 °C, a temperature-increase rate of 2.5 °C min^–1^, and a holding time of 2 h. The resulting pellets were ground using an agate mortar to obtain powdered CN. The obtained CN was dispersed in 20 mL of a water/ethanol 1:1 solution and washed with ultrasonic waves for 5 min. Subsequently, CN was combined with CdS using a precipitation method. The resulting CN powder (0.25 g) was dispersed in 100 mL of an ethanol solution prepared with 1.0 mol/L of sodium hydroxide, and 0.537 g of cadmium nitrate tetrahydrate was added and stirred for approximately 1 h. The powder was then ultrasonicated for 5 min. Subsequently, a solution of 0.415 g of sodium sulfide nonahydrate in 20 mL of pure water was slowly added dropwise to form a precipitate. The synthesized composite photocatalyst had a tCN:CdS ratio of 1:1 by weight. After stirring the solution for approximately 1 h, the precipitate was collected and washed with ethanol and pure water to remove impurities. The washed precipitate was dried, and the pellets were ground in an agate mortar to obtain a powdered tCN/CdS photocatalyst. All ultrasonication and stirring were done at room temperature without any gas substitution.

### Mo Photodeposition and Synthesis of TCM Photocatalyst

To synthesize TCM, we developed a novel method for reducing the Mo precursor in tCN/CdS under photoirradiation. Water was placed in a flask (30 mL), to which 0.0459 g of ammonium molybdate tetrahydrate was added and dissolved; 0.25 g of tCN/CdS was added to this and stirred in the dark for approximately 1 h to form a suspension. Subsequently, 20 mL of methanol was added to the flask, and photodeposition was performed via photoirradiation using an Xe lamp [Eagle Engineering, Japan] for 1 h. The precipitate was then collected and powdered; the powder was washed with pure methanol to remove impurities. The washed precipitates were dried and the pellets were ground in an agate mortar to obtain powdered TCM photocatalysts with a tCN:CdS:Mo ratio of 1:1:0.2 by weight; samples with different Mo weight ratios were prepared by varying the amount of ammonium molybdate tetrahydrate; 1:1:1 (TCM 1.0), 1:1:0.5 (TCM0.5), and 1:1:0.1 (TCM0.1) samples were prepared and compared to the 1:1:0.2 (TCM) samples. All ultrasonication, stirring, and photoirradiation were done at room temperature without gas substitution.

### Characterization

The crystal structure was measured at Cu Kα using an X-ray diffractometer (XRD) [RIGAKU, Japan]. The absorption properties are measured as the diffuse reflectance spectra (DRS) of powder using UV–vis spectroscopy [HITACHI, Japan]. The microstructures of the samples were photographed using scanning electron microscopy (SEM) [Carl Zeiss, Germany], and the elemental analysis was performed using energy-dispersive X-ray spectroscopy (EDS) [Burker, USA]. X-ray photoelectron spectroscopy (XPS) [JEOL, Japan] was used for the detailed valence and ionic state analyses.

### Hydrogen Sulfide Removal

The hydrogen sulfide decomposition performance was measured using the following method. First, after stabilization of the hydrogen sulfide generation in the permeator (GasTech), 0.05 g of the catalyst was placed in a quartz-glass reaction tube, and hydrogen sulfide diluent gas was steadily vented into the reaction tube at a concentration of 10 ppm and flow rate of 300 mL/min. Hydrogen sulfide was vented until the outlet concentration reached equilibrium, after which the light irradiation lamp was turned on to illuminate the catalyst for 3 h. During this period, the catalysts were exposed to light every 30 min; the outlet concentration was also measured every 30 min to analyze the decomposition performance. For light irradiation, an Xe lamp was equipped with a 400 nm cutoff filter, and only visible light of a constant intensity was irradiated onto the catalyst with a 20 cm opening from the light source to the reaction tube.

### Photocatalyst Cycle Test

Photocatalytic cycling tests were performed by collecting the samples after they had been used to decompose hydrogen sulfide. For the “washed” sample, the recovered catalyst was placed in a test tube, and 10 mL of a 5:1 water/methanol solution was added; the sample was then subjected to a 30 min wash in the dark. The “washed + light irr.” sample was washed for 30 min under light irradiation with an Xe lamp without any filter instead of washing the “washed” sample. These samples and the “blank” sample were placed in the reactor, and the hydrogen sulfide decomposition performance test was repeated.

## Results and Discussion

### Synthesis of TCM and Its Hydrogen Sulfide Removal

**Figure 1:**
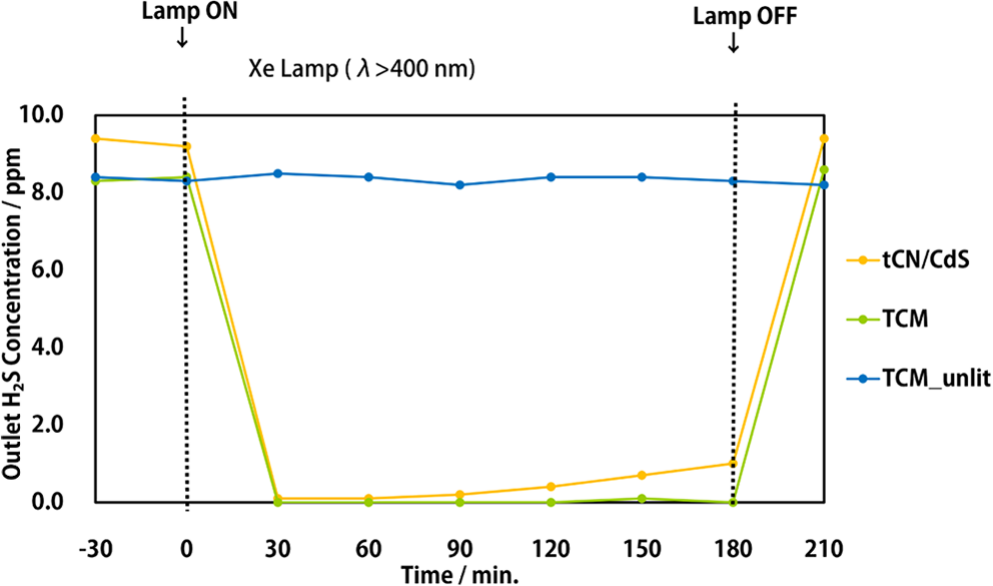
Hydrogen sulfide decomposition of photocatalysts; tCN/CdS: base hybrid photocatalyst with thiourea-driven carbon nitride and cadmium sulfide, TCM: molybdenum-loaded tCN/CdS, TCM_unlit: TCM sample for comparison without any light irradiation.

First, the results of the experiment comparing the hydrogen sulfide decomposition performance of the three types of samples, namely TCM without light irradiation (TCM_unlit), TCM with light irradiation (TCM), and tCN/CdS (raw material used for TCM synthesis), are shown in Figure 1.

For TCM without light irradiation, the outlet concentration of hydrogen sulfide became parallel at approximately 8.2 ppm; the concentration did not change significantly thereafter. TCM had a lower outlet concentration than tCN/CdS (9.4 ppm), indicating that the fine molybdenum particles supported on TCM partially reacted with hydrogen sulfide, causing the catalytic reaction to proceed in the dark and convert to molybdenum sulfide. Moreover, molybdenum is known as a thermocatalyst that reacts for sterilization and reduction; therefore, the equilibrium concentration was lower than the tCN/CdS one.

Conversely, in the sample in which the photocatalytic reaction proceeded by light irradiation 30 min after the equilibrium check, the hydrogen sulfide outlet concentration significantly decreased immediately after the start of light irradiation, and the hydrogen sulfide fed at 10 ppm decomposed to below the detection limit at 30, 60, and 90 min. In addition, while the hydrogen sulfide decomposition performance of tCN/CdS gradually decreased owing to poisoning, TCM maintained a high performance until 180 min and finally succeeded in reducing the outlet concentration to the lower detection limit within 180 min. The TCM sample turned dark green after the reaction compared to the sample before the reaction. Although this phenomenon was also observed in TCM without light irradiation, the TCM after light irradiation changed to a darker color, owing to the molybdenum particles in the catalyst reacting with the sulfide ions of the hydrogen sulfide that decomposed in the photocatalytic reaction and changed to molybdenum sulfide. Although molybdenum is partially sulfidized by ventilation alone, the hydrogen sulfide concentrated near molybdenum is presumably photoreduced by cadmium sulfide under light irradiation, and the molybdenum surface absorbs the sulfide ions and radical species generated in the process without further oxidation.

**Figure 2:**
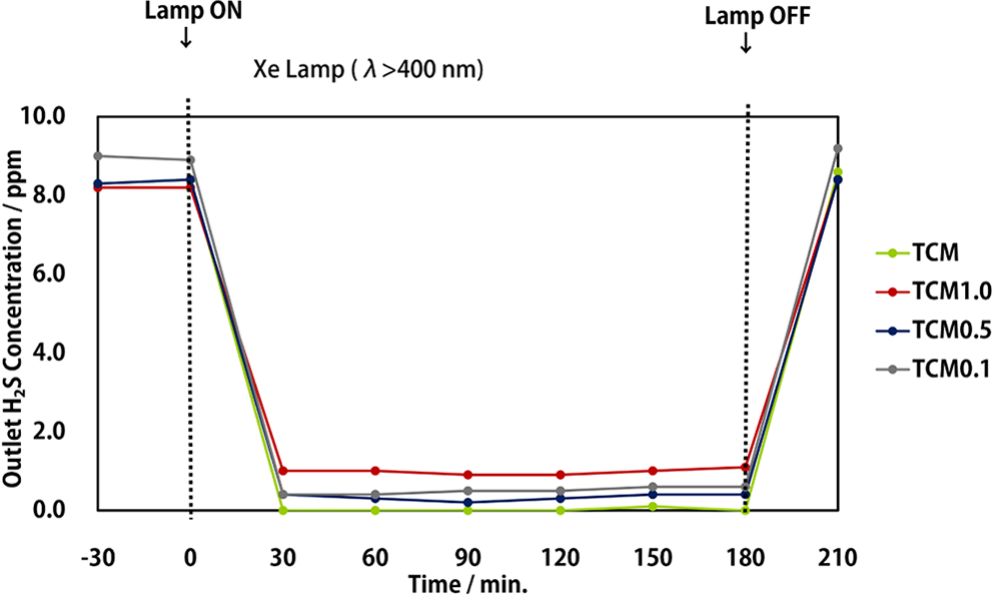
Hydrogen sulfide decomposition of each sample with different molybdenum loading ratios. tCN:CdS:Mo weight ratios are following; TCM1.0:1:1:1, TCM0.5:1:1:0.5, TCM0.1:1:1:0.1, contrast TCM: 1:1:0.2.

Figure 2 presents the experimental results of the hydrogen sulfide decomposition performance of each sample at different molybdenum loading ratios.

First, the performance degradation owing to poisoning, which was observed with tCN/CdS, could not be confirmed for all the samples, and nearly the same outlet concentration was maintained from the start to the end of decomposition. Therefore, molybdenum loading can prevent performance degradation owing to poisoning, even in insignificant amounts. Overall, the hydrogen sulfide decomposition performance was the highest for the TCM sample with a tCN:CdS:Mo ratio of 1:1:0.2, and the performance decreased with a higher or lower molybdenum loading ratio.

The performance of TCM1.0, which had the highest molybdenum loading ratio, was the lowest, whereas TCM0.5 and TCM0.1, which had the lowest molybdenum loading ratio, demonstrated only a slight increase in the outlet concentration compared to the TCM sample. The mediocre performance of TCM1.0 is considered to be owing to the excessive deposition of molybdenum interfering with the light absorption of the base material, tCN/CdS. The catalyst deposited on the surface of the photocatalyst absorbs light and is not involved in photoelectron excitation, which may have resulted in the mediocre performance of the photocatalyst. For TCM0.5, the hydrogen sulfide outlet concentration decreased further at 60 and 90 min after the start of light irradiation compared to the measurement point at 30 min. This may be because molybdenum is sulfated by the progress of the photocatalytic reaction. The decomposition efficiency of hydrogen sulfide is further improved owing to the increase in molybdenum sulfide, which acts as a photocatalyst.

**Figure 3:**
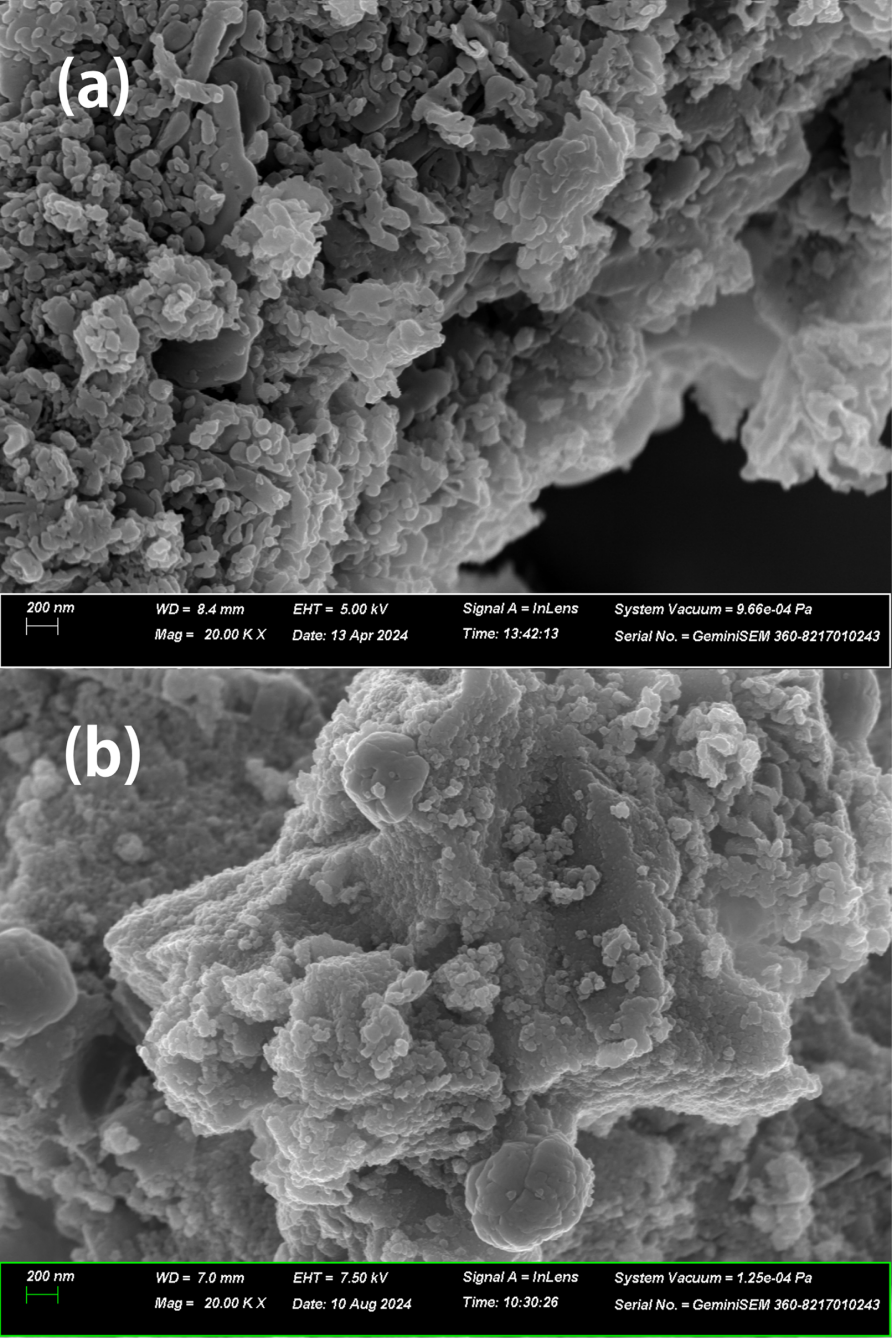
SEM images of photocatalysts; (a): tCN/CdS ×20k, (b): TCM ×20k. All SEM parameters are below the image.

The SEM images of photocatalysts are presented in Figure 3.

The porous stacked structure in tCN/CdS before the composite was well-retained in the TCM after molybdenum loading, indicating that cadmium sulfide was deposited on the tCN layer. In addition, the SEM images showed that the molybdenum spheres were supported in the TCM. The porous stacked structure likely contributes to the adsorption of hydrogen sulfide and increases the surface area with the reaction activity via light absorption. The molybdenum agglomeration contributes to the control of the sulfide ion reaction sites during the reaction, resulting in a low poisoning and high hydrogen sulfide decomposition performance.

**Figure 4:**
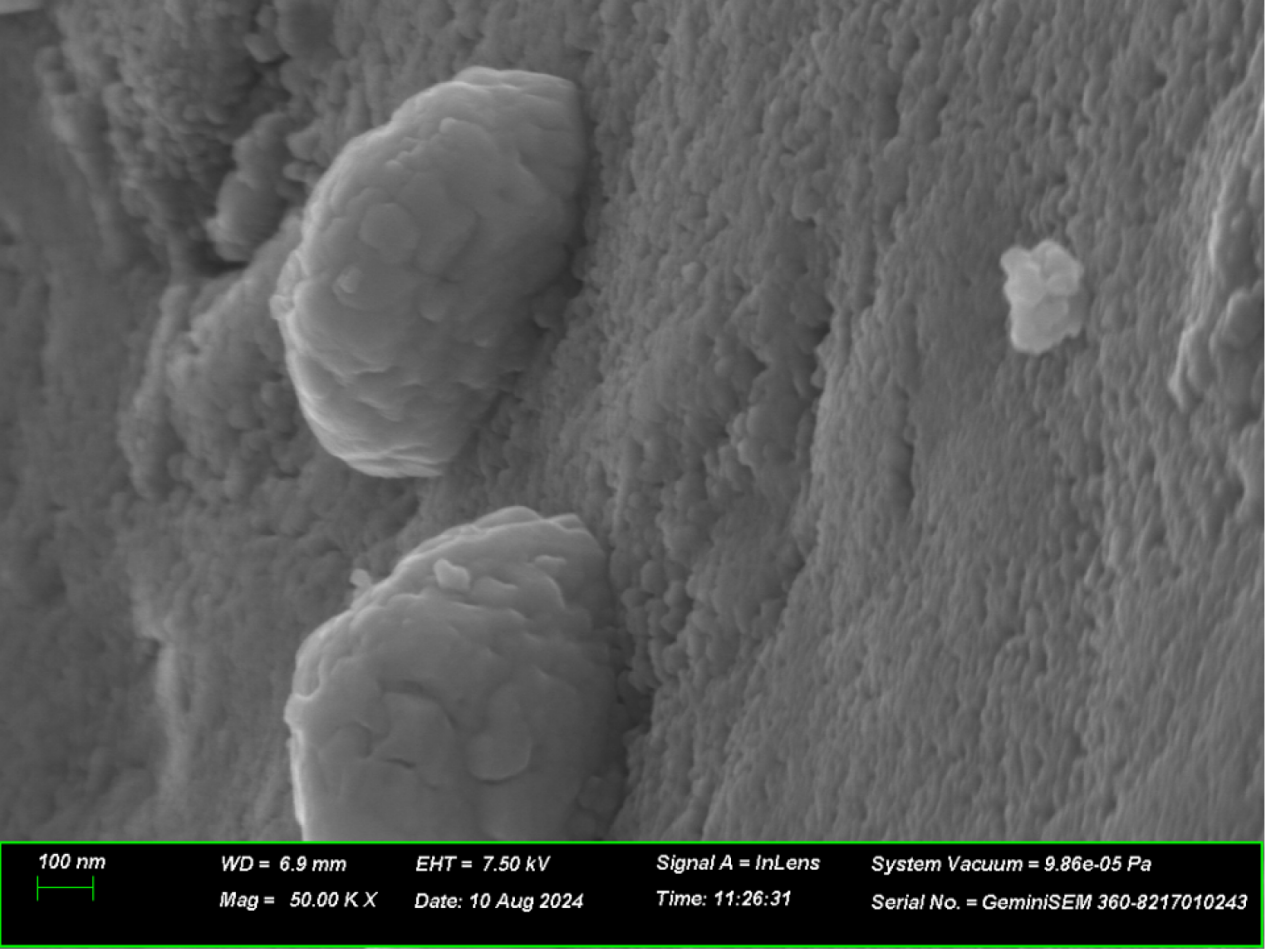
SEM image of molybdenum sphere on photocatalyst surface of TCM ×50k. All SEM parameters are below the image.

An enlarged image of the area (Figure 4) demonstrates that a large sphere is generated by the agglomeration of smooth surfaces on a rough surface, suggesting that the layered structure of Mo was formed by stacking and agglomeration.

**Figure 5:**
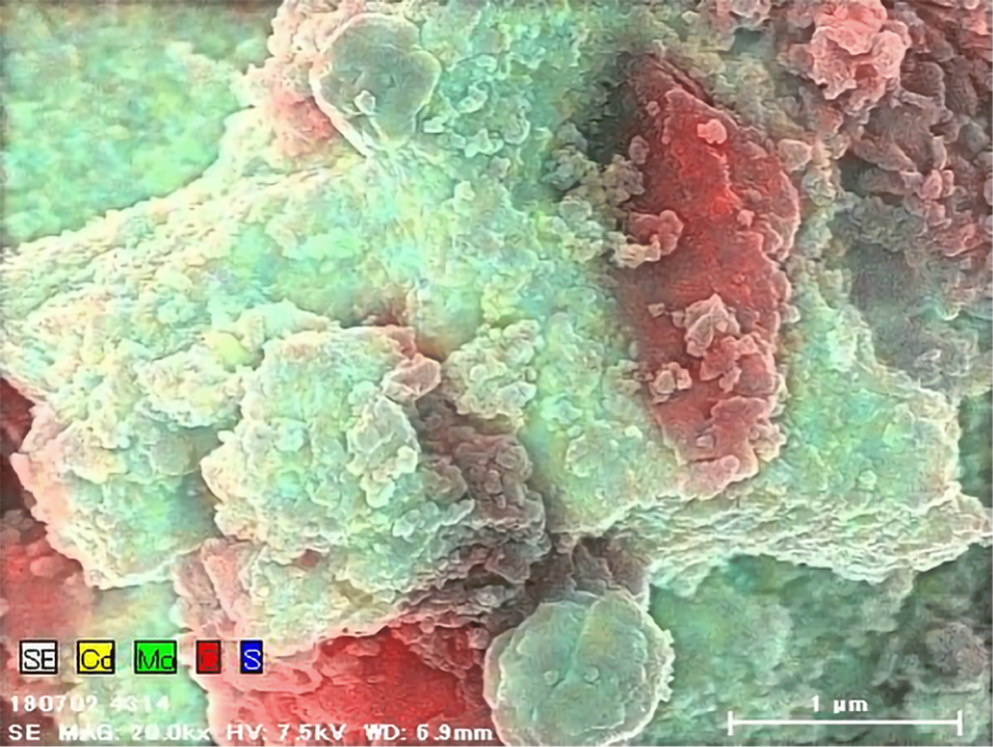
EDS survey mapping image of TCM ×20k. Elemental separate images are in Figure 6. EDS mapping was taken with these parameters: Voltage: 7.5 kV, Working Distance: 6.9 mm.

The EDS mapping images of TCM after compositing are shown in Figure 5.

**Figure 6:**
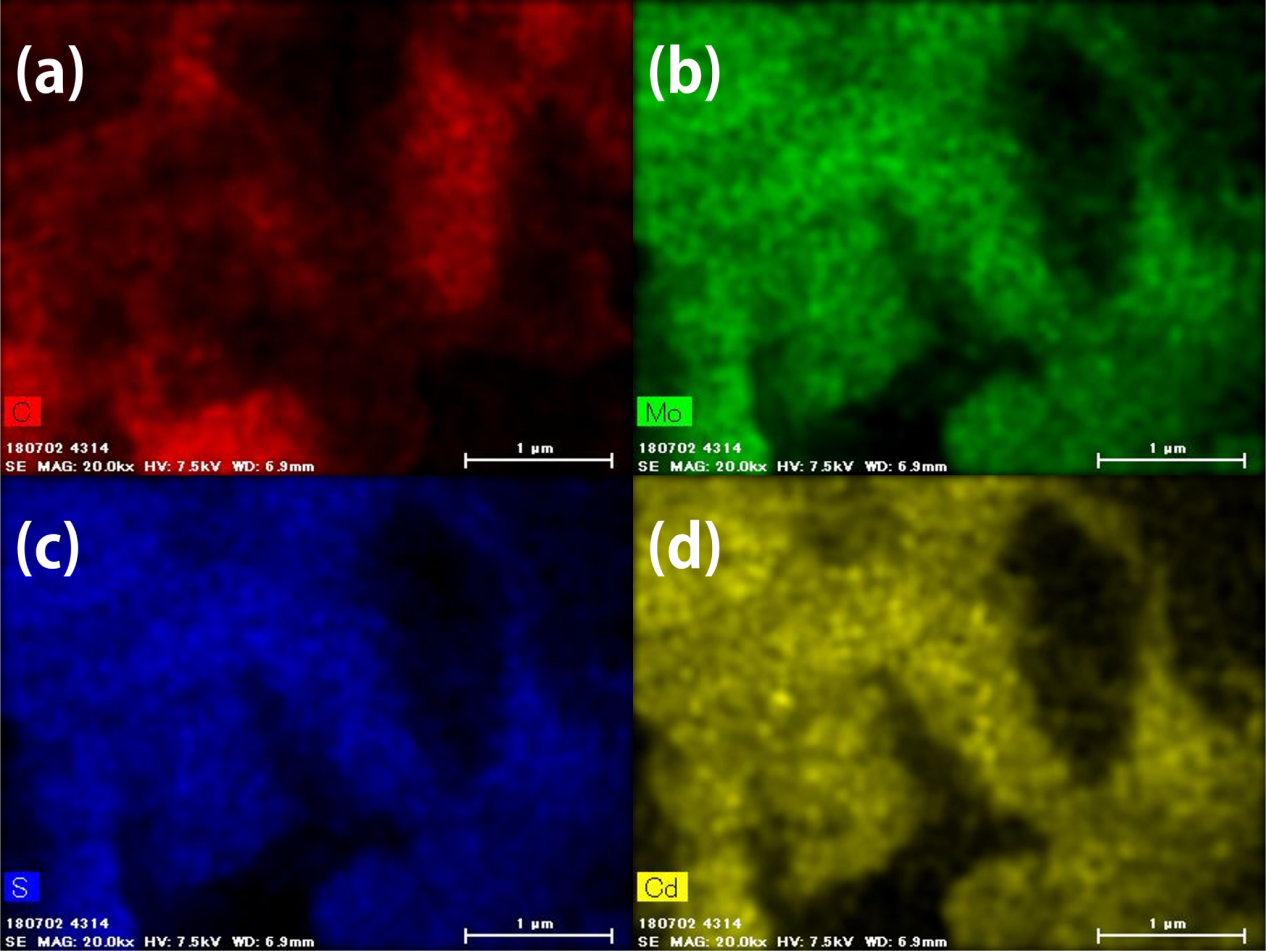
EDS mapping image of TCM ×20k; (a): carbon, (b): molybdenum, (c): sulfur, and (d): cadmium. EDS mapping was taken with the same parameters as in Figure 5.

The molybdenum (green), cadmium (yellow), and sulfur (blue) areas were almost identical, suggesting that molybdenum was loaded onto the cadmium sulfide surface (Figure 6). The carbon area (red) shows signals in different regions, suggesting that the tCN surface was partially covered with CdS, and molybdenum was deposited on the CdS. This may be because molybdenum was photocatalytically loaded from the precursor, and cadmium sulfide was responsible for the reduction reaction from the charge separation of the composite photocatalyst.

**Figure 7:**
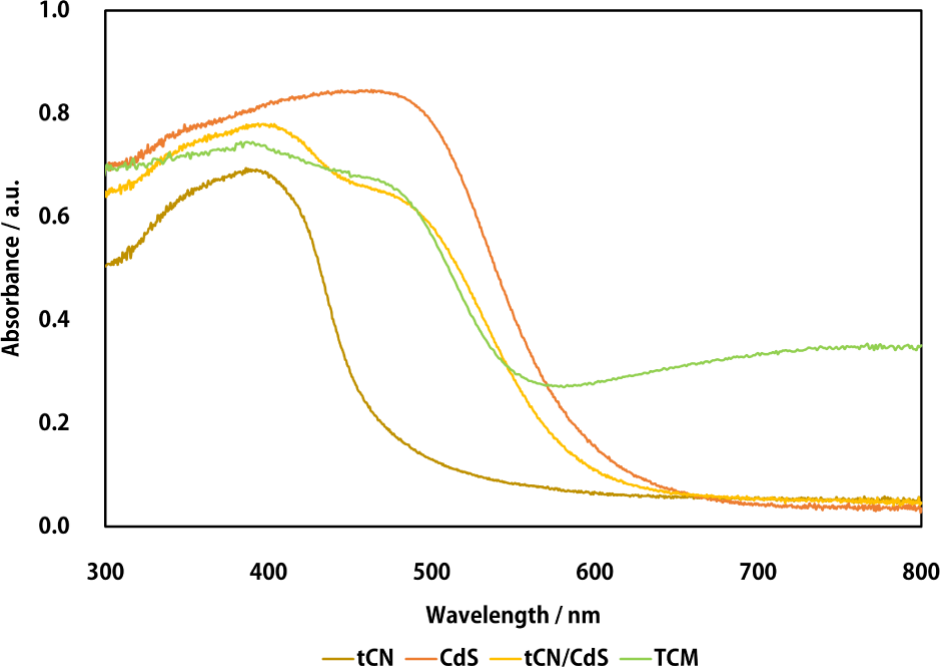
DRS of photocatalysts; tCN, CdS: ingredients of the hybrid photocatalyst, tCN/CdS: the composited hybrid photocatalyst, TCM: molybdenum-loaded tCN/CdS.

The results of the DRS measurements for tCN, cadmium sulfide, and tCN/CdS before compositing and for the molybdenum-loaded TCM samples are shown in Figure 7.

After molybdenum-loading, the tCN (approximately 400 nm) and CdS (approximately 500 nm) peaks were maintained. An increase in the peaks was observed in the absorption band above 600 nm. This was attributed to the wide-area optical absorption caused by the molybdenum support. This result is consistent with the fact that the samples slightly darkened after molybdenum loading. Although the molybdenum loading slightly reduced the light absorption of the photocatalyst substrate, the hydrogen sulfide decomposition performance improved, indicating that the molybdenum loading did not reduce the total performance of the sample.

**Figure 8:**
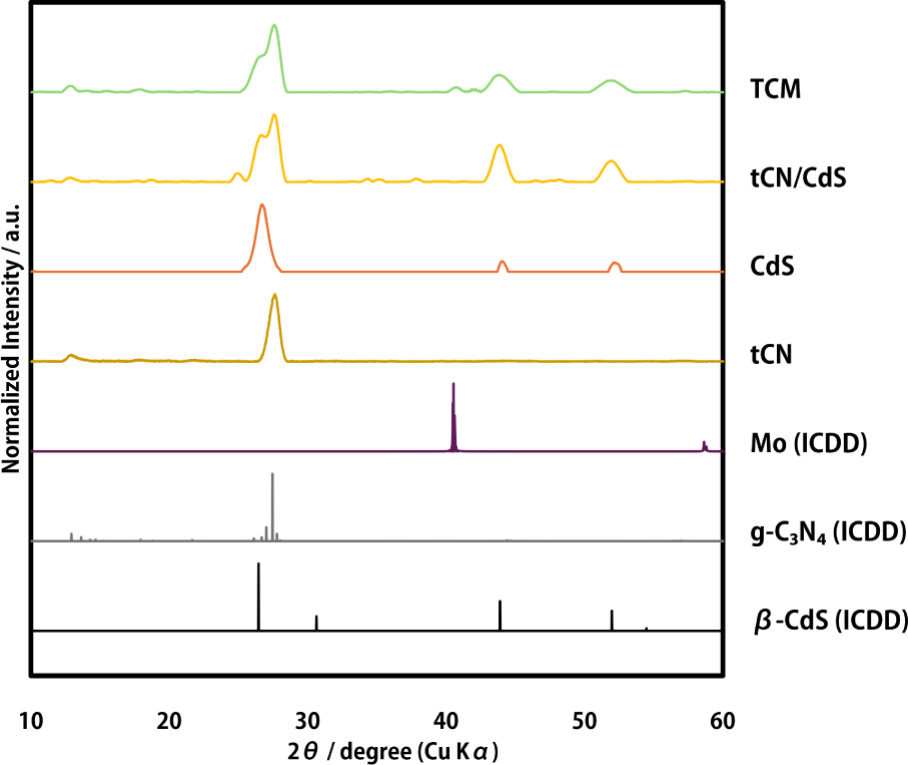
XRD Patterns of each photocatalyst and standards; TCM, tCN/CdS, CdS and tCN: synthesized sample in this research. Mo(ICDD): standard reference of ICDD-04-014-7435, g-C_3_N_4_(ICDD): standard reference of ICDD-00-053-067,β-CdS(ICDD): standard reference of ICDD-00-010-0454.

The results of the XRD characterization are shown in Figure 8. Only the TCM sample had a low yield per synthesis, so XRD was taken from a combined sample of five syntheses. The other samples were taken from one synthesis sample and measured.

For the TCM sample, the peaks for each catalyst were included and appropriately combined. In particular, a broad peak of about 40 degrees was detected for molybdenum. This suggests that trace amounts of molybdenum metal are deposited on the surface as small crystallites.

### Photocatalyst Cycle Test and Regeneration

**Figure 9:**
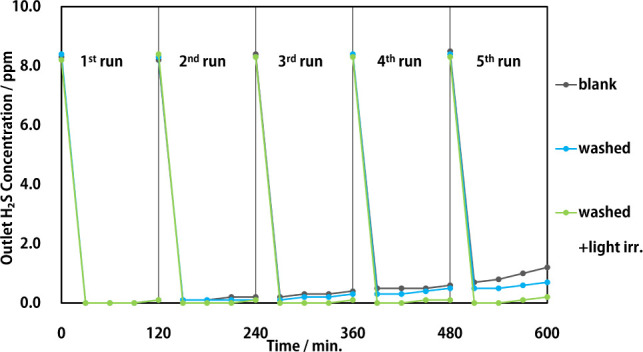
Photocatalyst-cycle test results: blank: unwashed sample, washed: washed sample on dark condition, washed+light irr.: washed sample with light irradiation.

The results of the photocatalytic cycling tests are presented in Figure 9. The horizontal axis indicates the total time of light irradiation. The sample to be washed was placed in the next run of light irradiation after washing.

The unwashed sample (blank) demonstrated a gradual decrease in performance after continuous light irradiation; the outlet concentration increased to approximately 1 ppm after 600 min. Conversely, the samples subjected to light irradiation during washing (washed+light irr.) recovered almost entirely after each wash. The final outlet concentration was approximately 0.2 ppm after 5 repeated tests. The wash-only sample (washed) did not significantly improve the catalytic performance, likely because the sulfated portions of the supported molybdenum were photocatalytically reduced to metallic molybdenum by washing with light irradiation. Generally, photocatalytic reactions in water generate hydrogen via water splitting; however, in the present reaction system, molybdenum sulfide was reduced instead of water, and the catalyst surface was chemically cleaned and regenerated.

**Figure 10:**
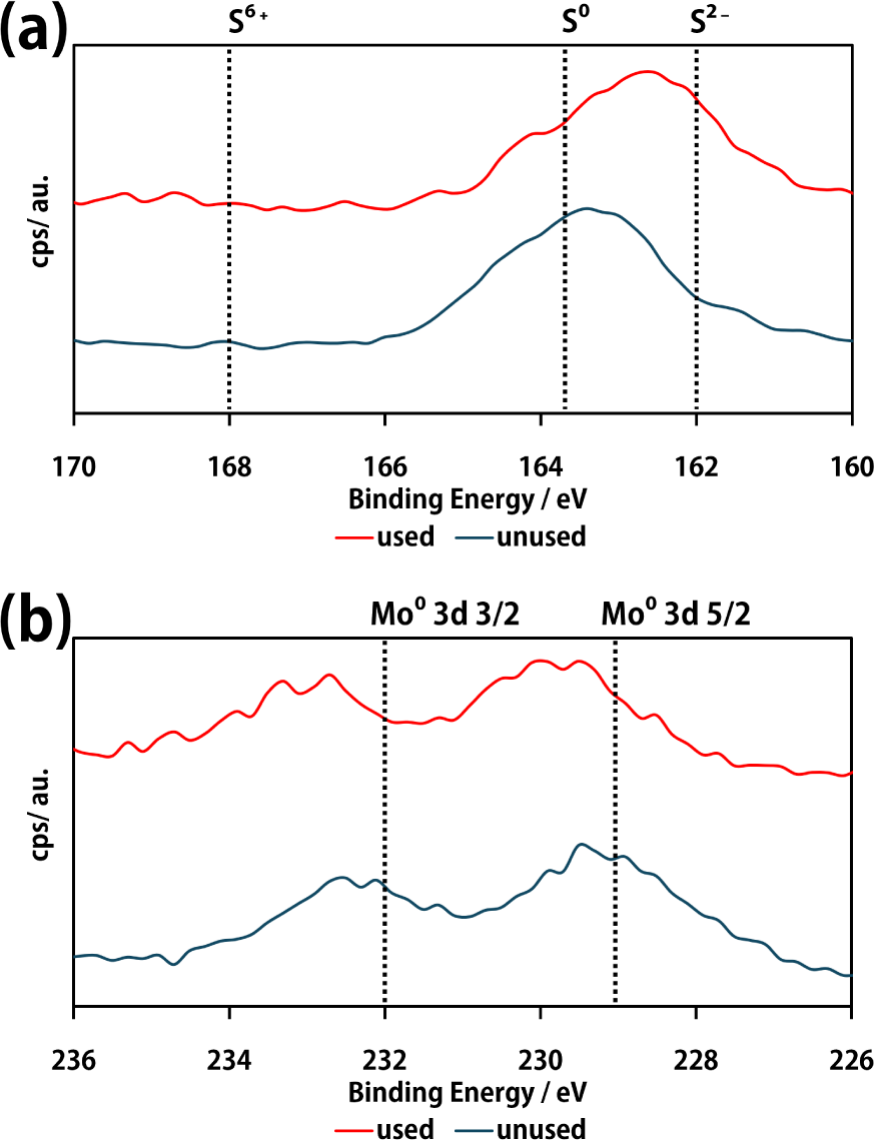
XPS Results before and after hydrogen sulfide removal; (a): S 2p 3/2, (b): Mo 3d 5/2 and 3/2.

The XPS results for sulfur and molybdenum before and after hydrogen sulfide removal are presented in Figure 10. For sulfur, the area around S 2p 3/2 (163.8 eV) was analyzed, whereas for molybdenum, the areas around Mo 3d 5/2 (229 eV) and Mo 3d 3/2 (232 eV) were analyzed.

First, the prominent peak shifted to lower energy for sulfur before and after the reaction, indicating an increase in the concentration of −2-valent sulfide ions. Furthermore, after the reaction, a slight increase in the peak intensity at approximately 168 eV was observed, which may be owing to the coverage of the catalyst surface by sulfate ions,^25^ which can be indicated as +6-valent sulfur. The increase in the +6S peak was minor compared to the increase in the +6S peak for the tCN/CdS photocatalyst observed in our previous study, suggesting that the TCM catalyst effectively suppressed sulfate ion formation. The two molybdenum peaks shifted by 1–2 eV before and after the reaction, suggesting that zerovalent molybdenum was partially transformed into molybdenum sulfide.

**Figure 11:**
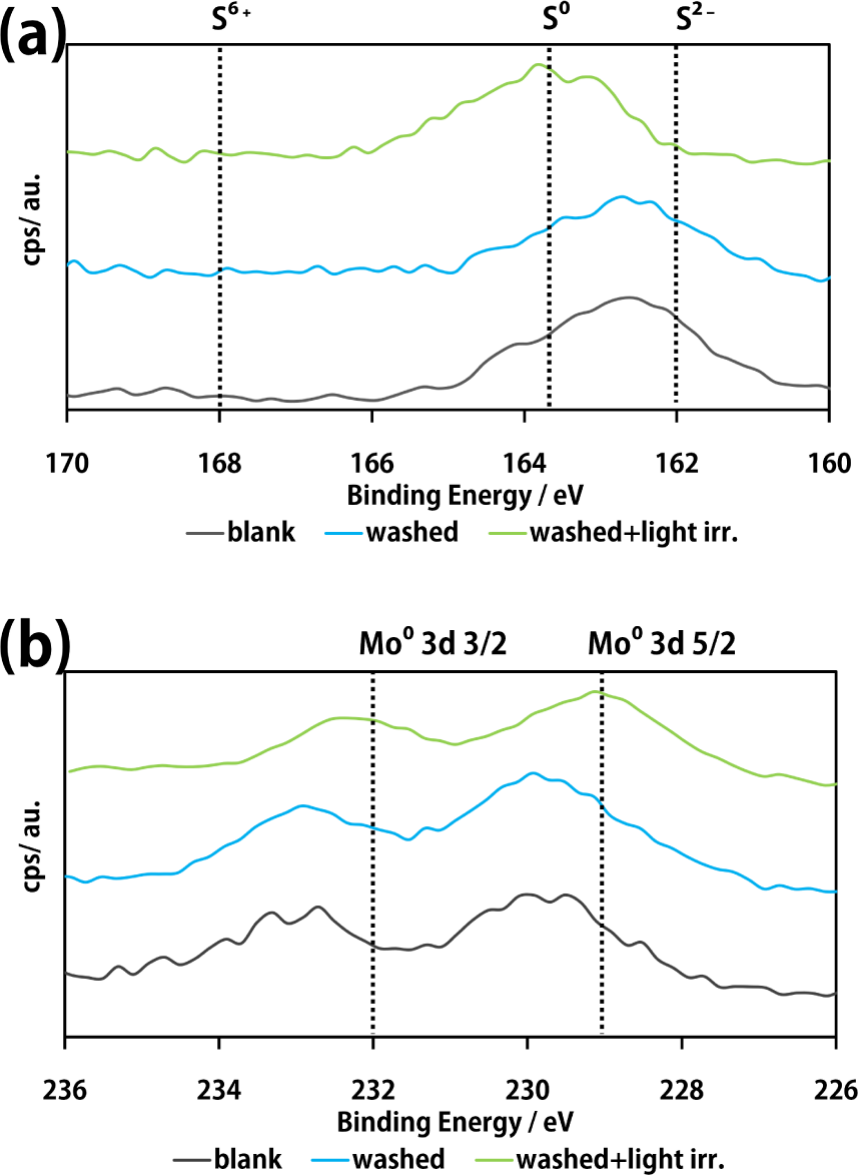
XPS Results of the cycle test; (a): S 2p 3/2, (b): Mo 3d 5/2 and 3/2. Samples of blank, washed, and washed+light irr. follow Figure 9.

Furthermore, the XPS measurement results for sulfur and molybdenum after five cycles for each blank, washed, and washed+light irr. samples are shown in Figure 11.

The washed+light irr. peak position of sulfur shifted to approximately 163 eV compared to that of the sample without light irradiation cleaning. This may be due to the relative decrease in the sulfur in molybdenum sulfide via photoreduction. For molybdenum, the peak shifted from 230 eV to approximately 229 eV after photoirradiation cleaning, suggesting that molybdenum sulfide was reduced. Based on these results, the photocatalyst proposed in this study achieved a photocycle in which the catalyst was partially sulfurized during the photolysis of hydrogen sulfide, and the catalyst can be regenerated by photocatalytic washing.

Thus, the TCM synthesized in this study achieved an efficient decomposition of hydrogen sulfide owing to its high photocatalytic activity and regenerative properties. In the synthesis stage, the tCN/CdS photocatalyst was selectively loaded onto the CdS surface, which was heavily affected by poisoning, using a novel photodeposition method in which the molybdenum precursor was reduced by photoexcitation in water to precipitate the metallic molybdenum. In addition, in the photocatalytic hydrogen sulfide vapor-phase treatment, the reaction proceeds as follows. Conducted hydrogen sulfide selectively reacts and decomposes on the electron-rich molybdenum surface excited by the photocatalyst, and the sulfide ions bond with molybdenum without further oxidation to form molybdenum sulfide. Molybdenum sulfide is also produced. Because the resulting molybdenum sulfide exhibits photocatalytic activity, the reaction proceeds on the molybdenum surface, and the photocatalyst-assistive catalyst interactions further enhance the activity. After the reaction progresses, molybdenum sulfide is reduced via light irradiation of the photocatalyst in the liquid phase, and the catalyst is regenerated to metallic molybdenum to complete the counter-poisoning of the catalyst, enabling the cycling of both hydrogen sulfide removal and catalyst regeneration in a reaction using light.

## Conclusion

This study introduced a molybdenum cocatalyst using a novel composite method utilizing photocatalytic activity. The synthesized photocatalyst exhibited a higher hydrogen sulfide decomposition activity and poisoning resistance than a stand-alone photocatalyst. It uses molybdenum as a cocatalytic site and acts as an electron-rich reduction site; molybdenum is partially converted to photocatalytically active molybdenum sulfide without producing sulfate ions that cause poisoning or only near the molybdenum, shown by the result of these characterization. This has maximally prevented the reduction of catalytic activity and is supported by the fact that the increase in sulfate ions observed in previous studies was significantly reduced in this sample.

In addition, washing with photoirradiation in water restored the catalytic performance, which was reduced. The high performance could be maintained despite another hydrogen sulfide decomposition. This may be because the partially sulfated molybdenum reverted to its metallic form via photoexcitation in water containing a reducing agent and was able to work as a cocatalyst once again. This research and the result would show that using this photocatalyst with high activity and long lifetimes will be critical for sustainable processes to decompose and treat various substances while reducing the environmental load. The photosupported molybdenum catalyst and the photocatalytic degradation-regeneration cycle in the gas-phase treatment proposed in this study are useful for various applications.

## Supporting Information Available

Additional previous research comparisons, schemes of the reactor, estimated reactions to H_2_S decomposition, photographs of catalysts, and characterization data (PDF)

## Abbreviations

CN: carbon nitride
tCN: thiourea-driven carbon nitride
TCM: thiourea-driven carbon nitride/cadmium sulfide/molybdenum photocatalyst
XRD: X-ray diffraction
SEM: scanning electron microscopy
EDS: energy dispersive X-ray spectroscopy
XPS: X-ray photoelectron spectroscopy
DRS: diffuse reflectance spectra
