# Supplementary material for: Photocatalytic Gas-Phase Hydrogen Sulfide Removal Using Mo Cocatalyst: Implementation of Counter-Poisoning Photocycle
Source: ACS Omega. 2025 Jan 29;10(5):4670–8. doi: 10.1021/acsomega.4c09395 (PMC11822519; doi:10.1021/acsomega.4c09395)
Supplement: Supplementary file 1 — ao4c09395_si_001.pdf [file ao4c09395_si_001.pdf]

Supporting Information

Photocatalytic Gas-Phase Hydrogen Sulfide Removal using Mo Cocatalyst: Implementation of Counter-Poisoning Photocycle

Tomofumi Katayama, Morio Nagata*

† Department of Industrial Chemistry, Graduate School of Engineering., Tokyo University of Science, 6-3-1 Niijuku, Katsushika-ku, Tokyo, 125-8585 Japan

*Corresponding Author: Morio Nagata

E-mails: 4223517@ed.tus.ac.jp (Tomofumi Katayama), nagata@ci.tus.ac.jp (Morio Nagata).

Table S1. Hydrogen sulfide decomposition activity compared to previous studies.

Catalyst	Outlet concentration [ppm]	H_2 reactivity [μmol/g_{cat}·h]	Condition	Ref.
g-C_3N_4/CdS/Mo (TCM)	0.00	145	10 ppm H_2S in air; 300W Xe Lamp, λ > 420 nm.	This study
CdS/zeolite	-	24000	H_2S in Na_2SO_3 aq; 240W Hg Lamp, λ > 400 nm.	[S1]
N-doped TiO_2	-	8800	H_2S in KOH aq; 300W Xe Lamp, λ > 420 nm.	[S2]
Anatase/TiO_2(B)	> 0.05	75	10 ppm H_2S in air; 18 mW/cm^2 254 nm UV Lamp.	[S3]
2% Au/SiO_2	-	18000	10 mL/min. pure H_2S gas, 13 W/cm^2 white light laser.	[S4]

These data were gathered from previous research. References are below; [S1]: Bai, X. F.; Cao, Y.; Wu, W. Photocatalytic decomposition of H_2S to produce H_2 over CdS nanoparticles formed in HY-zeolite pore, *Renewable Energy* **2011**, *36*, 10, 2589-2592. [S2]: Chaudhari, N.S.; Warule, S. S.; Dhanmane, S. A.; Kulkarni, M. V.; Valant, M.; Kale, B. B. Nanostructured N-doped TiO_2 marigold flowers for an efficient solar hydrogen production from H_2S, *Nanoscale* **2013**, *5*, 9383-9390. [S3]: Uesugi, Y.; Nagakawa, H.; Nagata, M. Highly Efficient Photocatalytic Degradation of Hydrogen Sulfide in the Gas Phase Using Anatase/TiO_2(B) Nanotubes, *ACS Omega* **2022**, *7*, 14, 11946-11955. [S4]: Lou, M.; Bao, J. L.; Zhou, L.; Naidu, G. N.; Robatjazi, H.; Bayles, A. I.; Everitt, H. O.; Nordlander, P.; Carter, E. A.; Halas, N. J. Direct H_2S Decomposition by Plasmonic Photocatalysis: Efficient Remediation plus Sustainable Hydrogen Production, *ACS Energy Lett.* **2022**, *7*, 10, 3666–3674.

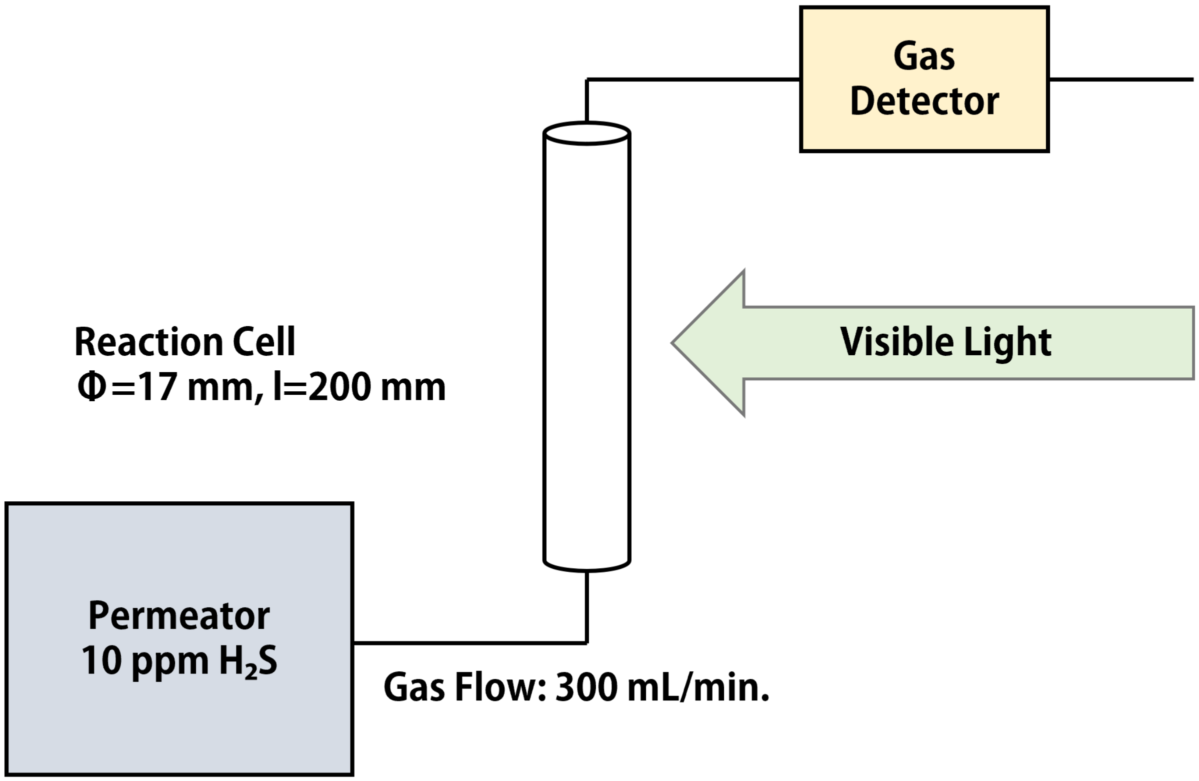

Figure S1. Schematic of the hydrogen sulfide decomposition reactor. Hydrogen sulfide was permeated to flow at a concentration of 10 ppm and a flow rate of 300 mL/min. Photocatalyst (50 mg) was put into the reactor cell and irradiated with visible light. Outlet concentrations were detected at the end of the reactor.

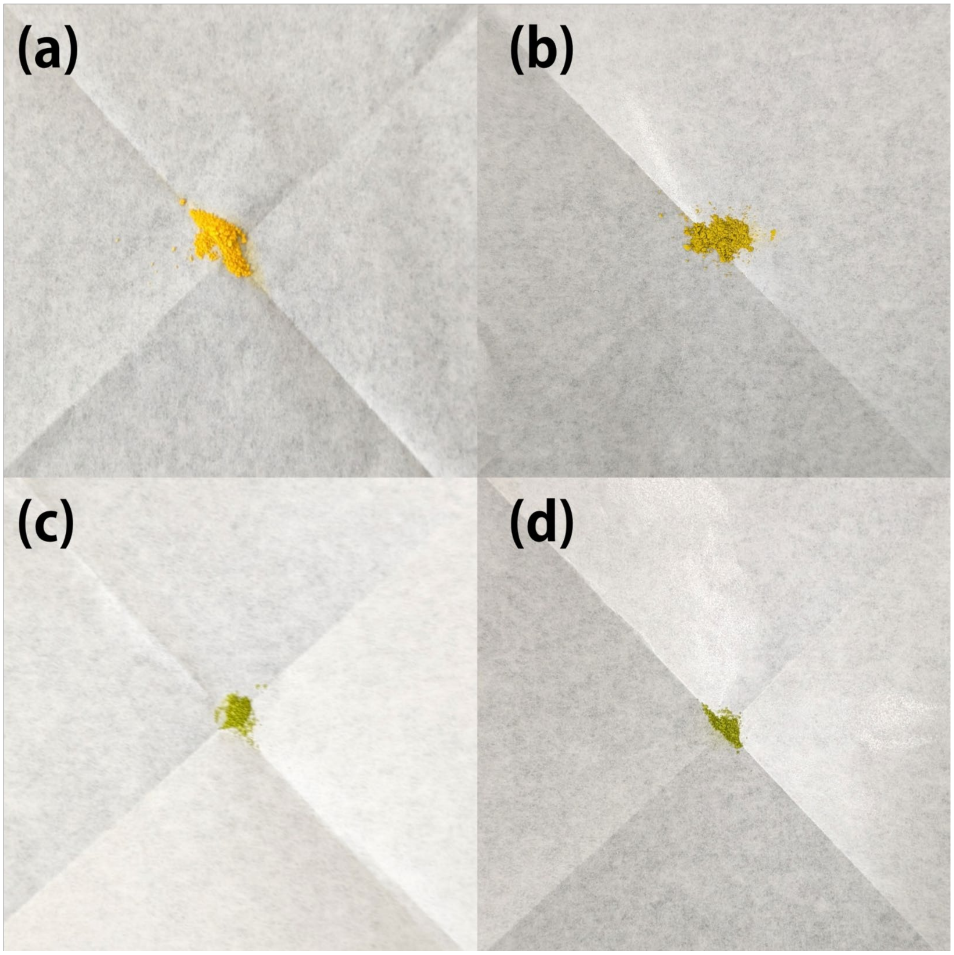

Figure S2. Image of synthesized and after-used photocatalysts; (a): tCN/CdS, (b): TCM, (c): TCM_unlit, TCM sample with 3 h H_2S exposure in dark condition, (d): TCM_lit, TCM sample with 3 h H2S exposure in light condition.

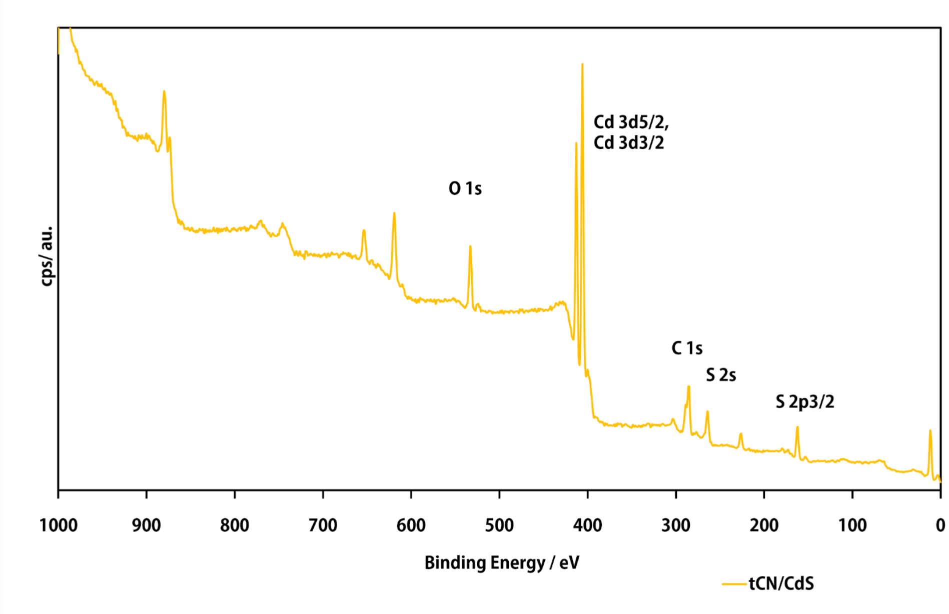

Figure S3. XPS survey spectra of tCN/CdS. Spotted elements from the catalyst surface by XPS are shown: O, Cd, C, S.

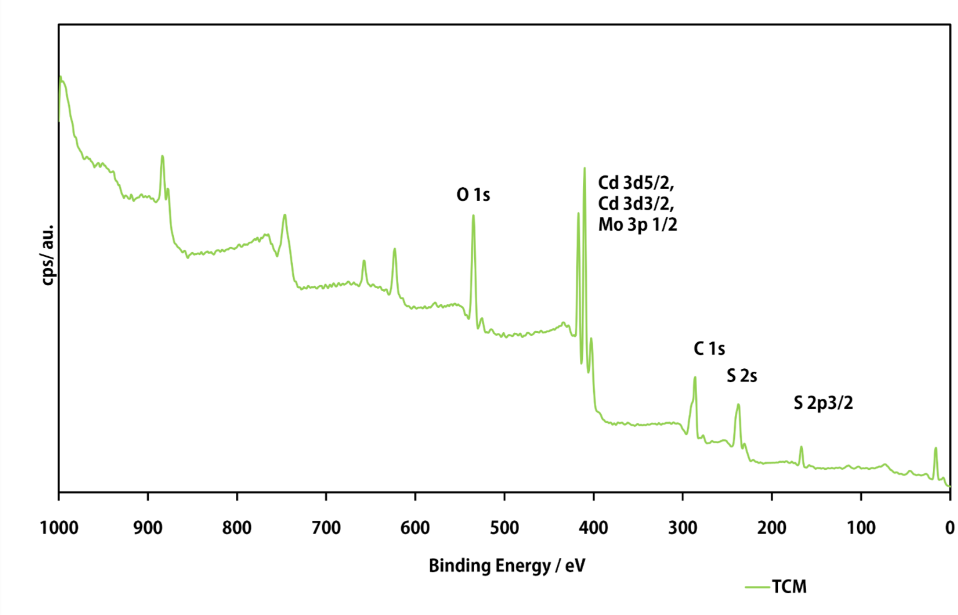

Figure S4. XPS survey spectra of TCM. Spotted elements from the catalyst surface by XPS are shown: O, Cd, Mo, C, S.

Table S2. XPS Surface Quantitative Survey of Unused and Used TCM Catalyst on Hydrogen Sulfide Removal Test.

Element	Unused TCM [wt%]	Used TCM [wt%]
C	74.45	65.42
S	5.72	8.19
Mo	8.50	8.98
Cd	11.33	17.41

These data were calculated from XPS quantitative spectra by weighting the peak ratios by the atomic weight of each element. The existence of sulfur and cadmium on unused TCM samples should be from cadmium sulfide, and Molybdenum should be a photodeposited molybdenum particle. The used sample's Mo:S ratio was 8.98:8.19 = 1:0.912, greater than the unused sample's Mo:S ratio; 8.50:5.72 = 1:0.673. It is considered that molybdenum sulfide was synthesized on the surface of the TCM catalyst with hydrogen sulfide adsorption and photocatalytic decomposition. (In this measurement, carbon tape was used to hold the powder in place, and since the carbon content ratio changes significantly due to its exposure, the Mo:S ratio was used for the evaluation.)

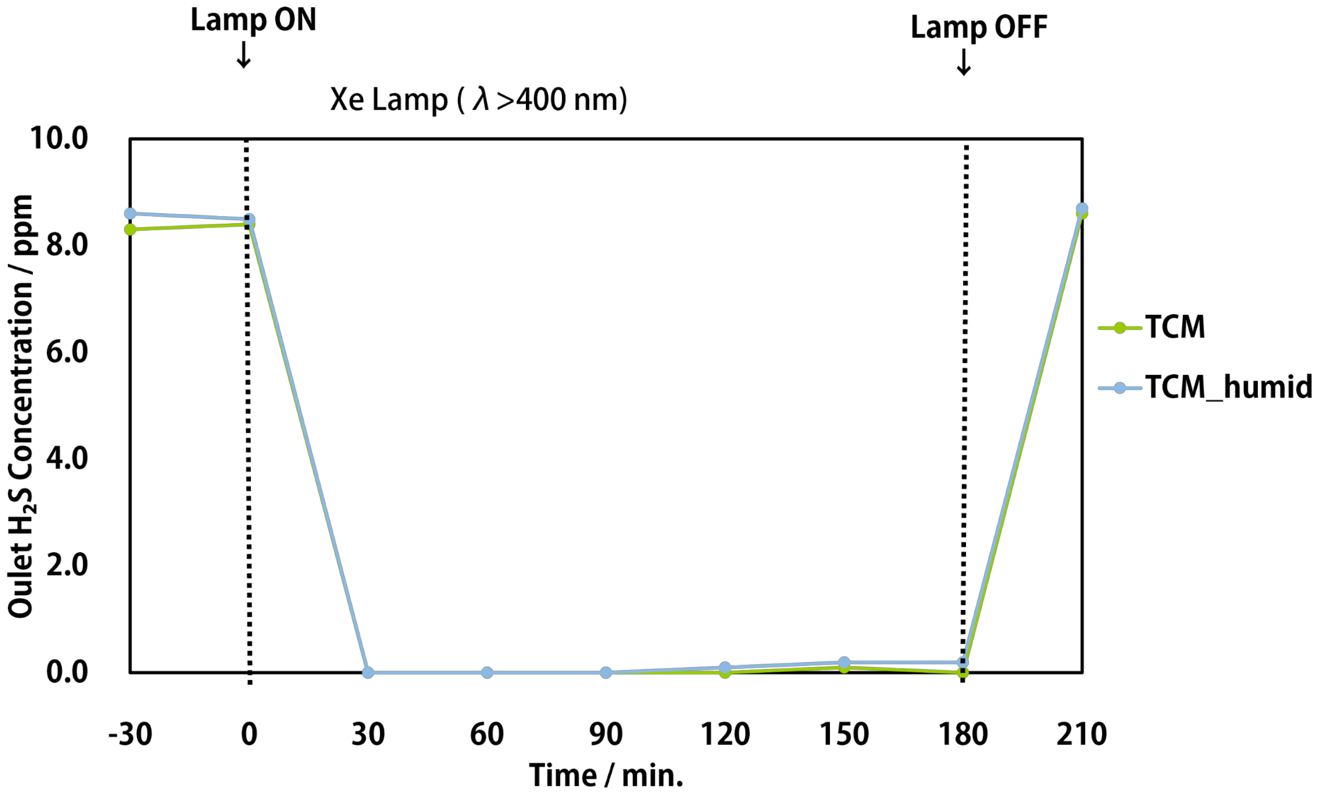

Figure S5. Hydrogen sulfide decomposition of TCM with and without humid air; TCM: molybdenum-loaded tCN/CdS without any air treatment, TCM_humid: TCM sample for comparison in high humidity; The experiment was conducted by preparing hydrogen sulfide gas with a relative humidity of approximately 80% by first bubbling air to dilute the hydrogen sulfide into a container of water.

The exit concentration in dry air was 0 ppm at 180 min, whereas in TCM_humid, it was 0.15 ppm. This photocatalyst could decompose hydrogen sulfide without any problem, even in a high-humidity environment, which was pointed out in previous studies as a possible obstacle to hydrogen sulfide decomposition using titanium dioxide.

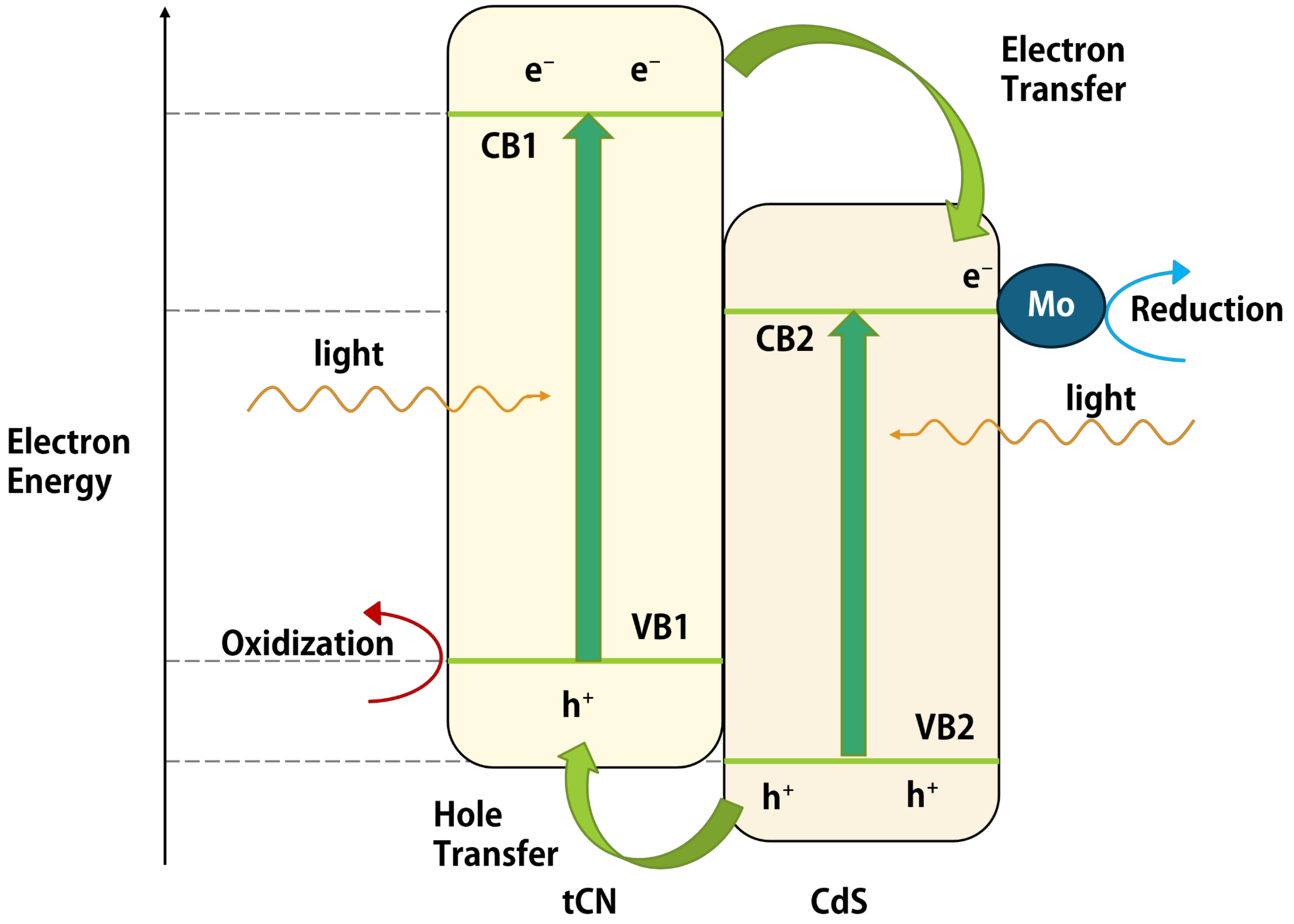

Figure S6. Estimated photoreduction reaction scheme. VB1 and CB1 are the valence and conduction bands of tCN; VB2 and CB2 are those of CdS. Molybdenum is an act of the electron career and hydrogen sulfide capture as a cocatalyst.
